# Subcortical Change and Neurohabilitation Treatment Adherence Effects in Extremely Preterm Children

**DOI:** 10.3390/brainsci14100957

**Published:** 2024-09-25

**Authors:** Susana A. Castro-Chavira, Claudia C. Gutiérrez-Hernández, Cristina Carrillo-Prado, Thalía Harmony

**Affiliations:** 1 Unidad de Investigación en Neurodesarrollo “Dr. Augusto Fernández Guardiola”, Instituto de Neurobiología, Universidad Nacional Autónoma de México, Santiago de Querétaro 76230, Mexico; castrochavirasa@inb.unam.mx (S.A.C.-C.); cgutierrez@comunidad.unam.mx (C.C.G.-H.); 2 Escuela Nacional de Estudios Superiores León, Universidad Nacional Autónoma de México, Guanajuato 36000, Mexico; ccarrillop@enes.unam.mx

**Keywords:** neurodevelopment, structural MRI, extremely preterm born, Katona neurohabilitation, longitudinal subcortical volumes

## Abstract

Extremely preterm birth entails an increased risk for multimorbidity and the prevalence of developmental deficits because this risk is negatively correlated to the number of gestation weeks. This work evaluated subcortical volume changes in children born extremely preterm who received Katona neurohabilitation, as well as the effects of subcortical volume and treatment adherence on their three-year-old neurodevelopment outcomes. Fifteen extremely preterm-born participants were treated from two months to two years old and followed up until past three years of age. The participants received Katona neurohabilitation, which provides vestibular and proprioceptive stimulation and promotes movement integration through the early, intensive practice of human-specific elementary movements. Subcortical brain volumes from magnetic resonance images were obtained at the beginning and after treatment. Also, treatment adherence to Katona neurohabilitation and neurodevelopment outcomes were measured. The results showed that absolute subcortical volumes increased after treatment; however, when adjusted by intracranial volume, these volumes decreased. Subcortical function inhibition allows cortical control and increased connectivity, which may explain decreased adjusted volume. Regression analyses showed that after-treatment hippocampal volumes had a discrete predictive value. However, treatment adherence showed a clear effect on mental and psychomotor neurodevelopment. Thus, the effectiveness of Katona neurohabilitation is constrained by treatment adherence.

## 1. Introduction

Every year, 15 million infants are born prematurely worldwide [1]. Preterm birth is highly correlated with brain damage and adverse neurodevelopment outcomes [2]. The more premature an infant is, the higher the risk of suffering a combination of multiple medical and neurodevelopment adverse outcomes, including brain damage and long-term neurocognitive sequelae [1,3]. Thus, extremely preterm newborns are at greater risk for neurological adverse outcomes. Individuals born extremely preterm have a 30-fold higher risk of cerebral palsy, a nine-fold higher risk of severe cognitive impairment, a seven-fold higher risk of epilepsy, and a four-fold higher risk of autism spectrum disorders [4].

Diffuse white matter injury underlies many of the long-term cognitive and sensorimotor deficits in children born preterm and is considered the main neuropathology in preterm infants [5]. White matter injury in this population is frequently accompanied by deviations and vulnerability in volumes of subcortical structures [6,7,8]. Infants born before 32 weeks of gestation have overall reduced brain volume, particularly in frontotemporal regions and hippocampi [9]. Multimorbidity including risk factors such as sepsis, bronchopulmonary dysplasia, intraventricular hemorrhage, steroid exposure, oxygen therapy, and sedation is frequently present in infants born extremely preterm. Moreover, these risk factors have been associated with smaller brain volumes [10].

Gray matter subcortical structures play a relevant role in sensorimotor, learning, emotion, and association loops [11]. Furthermore, the cortex in young children is immature and, consequently, subcortical processes are dominant. The cortex, basal ganglia, and cerebellum are crucial in typical brain development and neurodevelopment disorders. Cortico-subcortical networks are known to mediate the automatic and controlled processing in human cognition [12]. Anomalies in the regional specificity of cortico-striato-thalamo-cortical circuits may underlie the deficits observed in frontal executive dysfunction, behavioral problems including attentional deficit/hyperactivity disorder, and attention-related learning disabilities present in preterm children [13]. The subcortical structures are involved in motor control as well as in cognition and emotion processing [12], hence their relevance in the comprehensive study of the alterations produced by extremely preterm birth and the alternate neurodevelopment trajectories in infants treated with the neurohabilitation therapy.

While the early and accurate diagnosis and prognosis of brain damage in a newborn are essential, treatment to prevent sensory, motor, cognitive, and behavioral impairments, and ultimately economic, academic, and life quality issues, is decisive. Numerous intervention alternatives ranging from medication to physiotherapy, psychotherapy, and education are available to restrain brain damage and cognitive disability progression in preterm infants. However, the long-term effectiveness of early developmental intervention programs, provided post hospital discharge, to prevent motor or cognitive impairment in preterm infants compared to standard medical follow-up of these infants remains unclear [14].

The effective treatment of chronic conditions requires not only compliance but adherence, which implies the occurrence of dynamic and complex changes from many role-holders over long periods to maintain optimal development in infants [15]. Professor Ferenc Katona created a neurohabilitation method to both assess the neurodevelopment status and guide the treatment of newborns at brain damage risk starting as soon as possible after birth, taking advantage of the still ongoing neural plasticity [16,17]. Katona neurohabilitation therapy produces significant neurodevelopment improvements in infants and children with prenatal and perinatal risk factors, such as prematurity [18,19,20,21,22,23,24]. Katona neurohabilitation highly relies on the treatment adherence of primary care providers because they are the ones who administer the neurohabilitation treatment. Moreover, decisions to change recommendations, medications, and/or communication styles to promote the participation of the primary care provider depend on treatment adherence. There is no “gold standard” for measuring adherence behavior, and the use of a variety of strategies has been reported in the literature. The measurement of adherence provides useful information that outcome-monitoring alone cannot offer, but it remains only an estimate of the actual behavior of the primary care provider.

Considering these antecedents, we hypothesized that neurodevelopment outcomes are associated with subcortical volumes, the change in these volumes throughout development, and the amount and quality of neurohabilitation undergone, as measured by a treatment adherence proxy.

Three objectives were formulated to test this hypothesis. The first objective was to evaluate the differences in absolute and relative subcortical volumes between the beginning and after neurohabilitation. The second objective was to explore the associations between relative subcortical volumes, age at scan, treatment adherence, and neurodevelopment outcomes. Finally, the third objective was to evaluate whether subcortical volumes and/or treatment adherence may be used to predict neurodevelopment outcomes at three years of age.

## 2. Materials and Methods

### 2.1. Study Design

Fifteen children born extremely preterm (25–27 gestation weeks, GW; 53% female and 47% male) were selected from a wide sample of infants diagnosed with prenatal and/or perinatal risk factors for brain damage and followed up into childhood. These children were part of the longitudinal transdisciplinary research protocol of the Neurodevelopment Research Unit of the Institute of Neurobiology, National Autonomous University of Mexico [21]. The protocol includes newborn infants aged three months or less (corrected age) with prenatal and perinatal risk factors for brain damage referred by public hospitals in Queretaro, Mexico, or private practitioners. The exclusion criteria are genetic factors associated with brain damage, cardiovascular pathologies, brain malformations, and/or chromosomal aberrations. At arrival, the infants are evaluated by a neuropediatrician and screened with electroencephalographic, auditory and visual event-related potential, brain magnetic resonance imaging (MRI), nutrition, attention, neurohabilitation, rehabilitation, neurodevelopment, socioeconomic, and psychological evaluations and followed up until eight years of age. Before enrollment, primary care providers are invited to participate, a detailed description of the evaluation and treatment requirements is provided, and informed consent is obtained by a social worker [21,22].

The present work analyzes data regarding prenatal and perinatal risk factors, MRI subcortical volumes (acquired at the beginning and after neurohabilitation), treatment adherence, and neurodevelopment outcomes.

### 2.2. Prenatal and Perinatal Risk Factors

A neuropediatrician collected risk factor and diagnostic label data from the report generated by the hospital where each infant was born. Also, a complete neurologic evaluation was performed following the Amiel-Tison recommendations. The participants’ data are summarized in Table 1.

### 2.3. Katona Neurohabilitation Treatment

Katona neurohabilitation is a diagnostic and therapeutic tool based mainly on vestibular stimulation using different head and body positions. These actions also stimulate the visual, auditory, proprioceptive, and sensorimotor receptors. Katona described more than 40 different head and body positions (maneuvers) according to motor development.

The main characteristics of this treatment are (1) the initiation of the method as soon as possible after birth to be facilitated by the brain plasticity, (2) highly intensive stimulation for 3 to 4 periods of 45–50 min daily, (3) a prolonged duration of more than one year, preferably two or more years, and (4) capacitation of members of the family in the performance of the exercises for the treatment. At the beginning of the treatment, the intensive practice is based on genetically determined, human-specific elementary movements named by Katona “elementary neuromotor patterns”. These elementary neuromotor patterns fulfill two purposes, verticalization and locomotion. The vestibular receptors activate the vestibular nuclei in the brain stem, and their developing descendent projections from the vestibular nuclei to the spinal cord reach the motor neurons that activate efferent muscular processes. The ascendant projections of the vestibular nuclei activate the thalamus, the cerebellum, and the basal ganglia already myelinated at birth. This intensive stimulation allows the organization of motor control. When pathways to the cerebral cortex are myelinated, voluntary movements come into action [17,21,25]. In the case of brain lesions, this intense stimulation enhances plasticity and induces mechanisms to obtain proper motor behaviors.

The neurohabilitation treatment should begin as soon as possible after birth, before the corrected age of 2–3 months. After 6 months, the “elementary neuromotor patterns” disappear; therefore, neurohabilitation is not useful. At the initiation of treatment, first, the specialized physiotherapist evaluated muscle tone (passive and active), hemi body symmetry, attention, eye tracking, auditory monitoring, and neurological signs of alarm (thumb in fist, scissor gait, strabismus, irritability, and axial hyperextension, among others). Then, according to the diagnostic evaluation, the physiotherapist decided the infant-specific treatment plan and trained the primary care provider(s), usually the mother, on the administration of the appropriate maneuvers (positions and movements) to practice with the infant at home at least three times a day, every day. Each session consisted of the practice of 6–9 different maneuvers. Primary care providers attended training daily for the first three months and continued with follow-up visits during the first two years of life of the infant. The physiotherapist supervised and adjusted the treatment maneuvers during the monthly assessment/feedback follow-up visits. The primary care provider(s) administered the treatment at home; thus, the measurement of the actual frequency, duration, and quality of the maneuver practice in everyday life is not accurate. For this reason, treatment adherence was operationalized as the number of assessment/feedback follow-up sessions with the physiotherapist attended by the primary care provider with the infant (0–12 assessment/feedback sessions) [21,22,25].

### 2.4. Brain MRI

#### 2.4.1. MRI Acquisition

Infants’ brains were scanned during natural sleep and using ear protection. MRI acquisition was performed at the beginning and after treatment either with an Intera Phillips 1.0T (Philips Medical Systems, Best, The Netherlands) or a Discovery MR750 3.0T GE (GE Healthcare, Milwaukee, WI, USA) scanner. Ages at scan are shown in Figure S1.

Anatomical images acquired with the Intera scanner included axial and sagittal T1-weighted conventional spin echo (SE), with a repetition time (TR)/echo time (TE) = 405/15 ms, flip angle = 62°, 15 slices, slice thickness = 5 mm, matrix = 256 × 166, field of view (FoV) = 220 × 220 mm, and voxel size = 6.0 × 0.8 × 0.8 mm^3^; and axial and coronal T2-weighted SE, with TR/TE = 2600/150 ms, flip angle = 90°, 30 slices, slice thickness = 6 mm, matrix = 256 × 153, FoV = 200 × 200 mm^2^, and voxel size = 0.8 × 6.6 × 0.8 mm^3^.

The images acquired with the Discovery MR750 scanner used a 16-channel neurovascular head coil (HDNV). These images included coronal 3D T1-weighted SPGR, with TR/TE = 6/2 ms, flip angle = 12°, 392 slices, slice thickness = 1 mm, matrix = 224 × 224, FoV = 220 × 220 mm^2^, and voxel size = 0.8 × 0.5 × 0.8 mm^3^; and coronal 3D T2-weighted SE, with TR/TE = 2500/68 ms, flip angle = 90°, 196 slices, slice thickness = 1 mm, matrix = 224 × 224, FoV = 220 × 220 mm^2^, and voxel size = 0.8 × 1.0 × 0.8 mm^3^.

#### 2.4.2. MRI Individual Analyses

After formatting the images with the dcm2bids and dcm2niix tools [26,27], the MRI Denoising Package [28] for Matlab (version R2022a) was applied to improve the signal-to-noise ratio. Then, the images were resliced to a 1 × 1 × 1 mm^3^ voxel size using the mri_convert tool of the FreeSurfer software (version 7.0.2). The FreeSurfer pipeline was used to segment brains through the Aseg tool [29] in images of children older than two years. Images of two-year-old or younger brains were segmented using the Infant FreeSurfer pipeline [30]. Figure S2 shows an example of brain segmentations from images acquired at the beginning and after Katona neurohabilitation. Once extracted, the volume values were divided by the estimated total intracranial volume to control individual differences in head size. Both the absolute and the resultant relative volumes were used to perform statistical analyses. The subcortical volumes per hemisphere were utilized for the analyses.

### 2.5. Neuropsychological Screening

The Mental Development Index (MDI) and the Psychomotor Development Index (PDI) of the Bayley Scales of Infant Development, Second Edition (Bayley-II) [31], were used to evaluate the neurodevelopment outcomes at three years of age.

### 2.6. Statistical Analyses

The SPSS v15.0 software was used to perform the statistical analyses. First, variable normality was assessed with Kolmogorov–Smirnov tests. Because several variables did not show normal distributions, Wilcoxon signed-rank tests were applied to evaluate differences in subcortical volumes at the beginning compared to after the treatment (*p* < 0.05).

Then, Spearman’s partial correlations, controlled by sex, were performed to evaluate the associations between subcortical relative volumes, age at first scan, time between scans, treatment adherence, and neurodevelopment outcomes (*p* < 0.05; uncorrected).

Finally, hierarchical regressions were performed to search for the combinations of variables that best predicted MDI and PDI scores (*p* < 0.05). The variables were introduced in two stepwise blocks: (1) age at first scan, time between scans, sex, and treatment adherence; and (2) subcortical relative volumes.

## 3. Results

### 3.1. Prenatal and Perinatal Risk Factors

Extremely preterm infants frequently show several comorbid prenatal and perinatal risk factors. In our sample, maternal infections (66.7%) and early membrane rupture (53.3%) were the most frequent maternal risk factors. Regarding the infant risk factors, neonatal sepsis (86.7%), hypoxic–ischemic encephalopathy (60.0%), and intraventricular hemorrhage (60.0%) were the most frequent. These extremely preterm children spent an average of 67.1 days hospitalized (standard deviation of ±21.3 days) and 40.9 days with ventilation therapy (standard deviation of ±37.4 days) (Table 1).

### 3.2. Subcortical Volume Change

Regarding the subcortical volume differences at the beginning and after treatment, the absolute volumes showed significant bilateral increases in the amygdala, caudate, hippocampus, putamen, total subcortical gray matter, cortex, white matter, and total intracranial volume after treatment compared to the beginning of treatment, while the pallidum and thalamus had non-significant changes (*p* < 0.05; Table 2). However, relative volumes, calculated by dividing the volume of each structure by the total intracranial volume, were significantly smaller (*p* < 0.05) after treatment compared to the volumes at the beginning of treatment, except for the right amygdala, both hippocampi, and white matter (*p* < 0.05). The right amygdala, right hippocampus, and white matter relative volumes tended to increase while the left hippocampus relative volume tended to decrease (Table 3).

### 3.3. Correlations between Subcortical Volumes, Treatment Adherence, and Neurodevelopment Outcomes

Spearman’s correlations controlled by sex allowed us to explore the associations between subcortical relative volumes, age at first scan, time between scans, treatment adherence, and neurodevelopment indices. Correlations of treatment adherence with age at first scan; Bayley’s Mental Developmental Index (MDI, Figure S3); Psychomotor Developmental Index (PDI, Figure S3); and right pallidum, cortex, and white matter relative volumes at the beginning of treatment were found. At the beginning of treatment, age at first scan negatively correlated with the bilateral amygdala, hippocampus, pallidum, putamen, thalamus, and subcortical gray matter. MDI positively correlated with the white matter relative volume at the beginning of treatment and PDI negatively correlated with the left putamen relative volume at the beginning of the treatment. MDI and PDI were correlated, also (uncorrected *p* < 0.05; see Table S1).

### 3.4. Neurodevelopment Outcome Predictors

Hierarchical regressions were performed to assess the predictability of the neurodevelopment indices, MDI, and PDI. The best model obtained to predict MDI (R^2^ = 0.598, *p* = 0.004) included treatment adherence and the relative volume of the right hippocampus. For PDI, the best model (R^2^ = 0.543, *p* = 0.009) used treatment adherence and the relative volume of the left hippocampus as predictors (Table 4).

**Figure 1:**
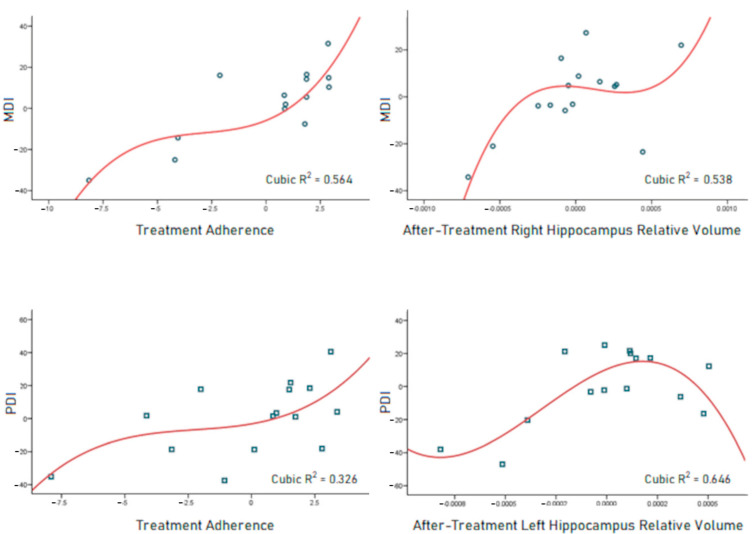
The hierarchical regression analyses yielded partial regression graphs for the predictors of mental (MDI; **top**) and psychomotor (PDI; **bottom**) development indices. Cubic curves, in red, yielded the best adjustment in every case (confidence interval of 95%). MDI cases are represented with blue circles and PDI cases are represented with blue squares.

The partial regression graphs generated from the hierarchical analyses showed that cubic equations yielded the best adjustment to predict MDI and PDI scores (Figure 1).

## 4. Discussion

Every year, 200,000 preterm infants are born in Mexico [32]. Though there are no reports on the number of extremely preterm infants in Mexico, a study carried out between 2015 and 2017 found that 7% of the preterm infants studied were born extremely preterm [33]. Thus, a gross estimation based on the previously mentioned publications results in approximately 14 000 extremely preterm infants born in Mexico every year.

The extremely preterm infants in this sample showed combinations of several prenatal and perinatal risk factors, which reflects the complexity of effects and interactions affecting the brain and its function in these children [4,10,34,35]. Maternal infections and neonatal sepsis together were present in 60% of the sample, which may be a relevant factor resulting in extremely preterm birth, as proposed by other authors [36,37]. Neonatal brain damage signs such as hypoxic–ischemic encephalopathy and intraventricular hemorrhage were also present in most of the participants, with occurrence of 60% for each, which is consistent with previous findings [36].

The results of treatments designed to reduce the sequelae of brain damage-related injuries and deficits that accompany preterm birth are limited. A systematic meta-analysis evaluated the results of 25 randomized and quasi-randomized studies of early interventions (before 12 months of age) which included 3615 participants. The interventions studied included the Bobath neurodevelopmental treatment, physical therapy, sensory stimulation (auditory, visual, tactile, and kinesthetic), motor exercise routines, psychological counseling, babywearing and/or educational sessions, and parenting counseling. The authors found that these interventions improved cognitive outcomes through preschool age, with modest improvements in motor outcomes during infancy and little evidence of cognitive or motor effects through school age. However, due to variations in study characteristics, comparisons between interventions were not made [14]. The aforementioned Bobath neurodevelopmental treatment is the most frequently used intervention in infants with neurodevelopment deficits. Bobath treatment and the Vojta method are generally applied when signs and symptoms of brain damage are present and are, therefore, neurorehabilitation therapies. On the other hand, the neurohabilitation therapy designed by Katona consists of the early diagnosis and treatment of brain damage, before 3 months of age. A study of 262 infants treated with Katona neurohabilitation therapy showed that over 40% and 69% of extremely to very preterm infants achieved normal scores on the MDI and PDI Bayley scores, respectively, assessed at 24 months (about 2 years) of age. Furthermore, although a very low percentage of infants born after 31 weeks had scores indicative of delay on MDI and PDI, over 80% of these infants had a normal PDI score [22]. On the other hand, a report comparing neurodevelopment outcomes in a group treated with neurohabilitation during the first two years of life and an untreated group both assessed between 6 and 8 years of age showed that the neurohabilitation group had a higher percentage of infants with normal neurodevelopment outcomes compared with the untreated group (90% vs. 38%). Among the children who presented normal MRIs (without apparent pathology), the treated group showed a higher percentage of normal neurodevelopment outcomes than the untreated group (100% vs. 50%), although differences between the groups were not significant [23]. Finally, another longitudinal study comparing the neurodevelopment treatment outcomes of Bobath and Katona neurohabilitation therapy in premature infants with perinatal brain damage showed that the group treated with neurohabilitation therapy had better neurodevelopment outcomes assessed after five years of age than the group treated with the Bobath method with equivalent starting time and treatment intensity [19].

Regarding brain structural changes, the total intracranial volume showed logarithmic-shaped growth (Supplementary Figure S4), as previously found for whole-brain growth in infants with typical development [38,39]. Decreasing total intracranial volume in the extremely preterm born toddlers, as occurred in one of the participants, may be associated with the dysmaturational events associated with preterm birth [40]. However, as reported elsewhere, reduced intracranial volume in preterm infants may catch up later in childhood [38]. A previous lifespan study from 3 to 90 years of age shows larger intracranial and subcortical volumes for healthy individuals; only the right amygdala volume in this study is similar to those found elsewhere [38,41]. However, no correlations or associations as a predictor of neurodevelopment were found for this volume.

When the subcortical volumes were compared between the beginning of and after treatment, increased sizes in most of the structures, except the pallidum and thalamus, were observed. The observed increases are expected due to developmental evolution, in accordance with Dima et al. [41]. On the other hand, other studies have found that children born preterm show lower brain volumes that catch up with age, which may explain the growth delay observed in our sample. The study by Alex et al. [38] found that maturation asymptote in the globus pallidum is reached at 5 years of age, while the thalamus has the most protracted growth trajectory, reaching maturation by 7.4 years of age. Because only five among the thirty magnetic resonance images were scanned after five years of age, protracted development cannot be inferred in our sample. On the other hand, the subcortical volumes divided by the total intracranial volume showed significant decreases, except for the right amygdala, bilateral hippocampus, and cerebral white matter. These findings agree with the reports by Østby et al. [42], Choe et al. [39], and Dima et al. [41]. Specifically, the amygdala has been found to develop early, reaching its volume asymptote by 3.3 years of age. In our sample, the median age for the after-treatment scans was 3.0 years old, with the right amygdala showing a tendency of increased volume and, thus, a growth pattern different from that of the left amygdala. Hemispheric asymmetries in the amygdala have been reported, with the right amygdala preferentially processing pain, fear, negative emotional valence, and visuo-spatial stimuli and showing a larger volume than the left in older children and adults [38,43,44]. Regarding the hippocampus, a maturation peak at 4.6 years has been reported, only preceded by the amygdala, which is consistent with our results [38].

Extremely preterm children are born around the beginning of the third trimester of gestation when thalamocortical axons depart subplate neurons and enter the cerebral cortex. Also, callosal and corticocortical axons enter the subplate and GABAergic neurons migrate into cerebral white matter during this period. The rapidity and complexity of cellular events during the third trimester of gestation underlie their vulnerability to perturbations. The principal components involved include the oligodendroglial lineage, especially the preoligodendrocyte, cerebral white matter axons, subplate neurons, the cerebral cortex, the thalamus, and basal ganglia [40]. At this stage, there is also an increased susceptibility of gray matter [35]. The underlying mechanism of injury involves an initial insult to the vulnerable, developing fetal brain that is usually either of hypoxic–ischemic, hemorrhagic, or infectious nature and sets off a cascade of events leading to further brain injury [45].

As an infant develops into a toddler and later into a child, the emergence of motor skills depends on the inhibition of the subcortical nuclei of the brain resulting from the maturation of the motor cortex [46]. Thus, top–down mechanism enhancement is consistent with increased cortical development at the expense of subcortical development reduction. Specifically, the pallidum is involved in voluntary movement regulation; its inhibition is required for dopaminergic signaling from the motor cortex to take over [46]. Regarding the thalamus, a limitation of this study was that the estimation of volumes was not accurate in all the subjects, as measured by the software for infant brains. However, this pipeline used showed better results than standard methods either informed by T2-weighted images or considering the presence of large lateral ventricles. Coincidental results were obtained in a study analyzing the developing Human Connectome Project sample, where bilateral growth decreases in the pallidum and thalamus were found during the second semester of life [47].

The growth rate trajectory of intracranial and subcortical volumes is faster in the first 1000 postnatal days and slower after this period. Highly rapid growth in the first few years of life make infant and young child brains especially vulnerable to environmental insults, such as preterm birth, but also especially responsive to interventions such as Katona neurohabilitation [38].

It is relevant to consider that the absence of MRI clinical signs of brain damage in extremely preterm children is not necessarily consistent with the absence of developmental disorders. Infants with no or mild abnormality in cerebral white matter as evinced by conventional MRI still exhibit neurological disability. Several studies report cognitive scores between 85 and 93 for infants (born at less than 28 to 30 weeks of gestation) with no or mild white matter injury. A study of 480 extremely preterm infants reported that 20% of infants with no apparent white matter injury by conventional MRI had cognitive scores less than 85 [40].

In our sample, four out of fifteen participants (less than 27%) presented with moderate-to-severe mental delay and none with mild delay, as measured by 3-year-old MDI score, while the Victorian Infant Collaborative Study (VICS) group reported 32% with mild cognitive delay, 12% with moderate delay, and almost 4% with severe delay at 2 years of age. The Swedish EXPRESS cohort reported 24% of children exhibited mild delay, 5% with moderate delay, and 6% with severe delay at the age of 2.5 years [14,48].

Regarding the psychomotor function, four showed moderate-to-severe delay and one showed mild delay out of fifteen (less than 27% and 7%, respectively), as measured by 3-year-old PDI score. In comparison, reports on extremely preterm toddlers using PDI have found moderate-to-severe motor delays in a 31–71% range [49].

To search for predictors for neurodevelopment outcomes, regression models were built. The main predictor of both MDI and PDI was treatment adherence. In addition, the hippocampal development after treatment allowed improved prediction models which enhanced its relevance for the neurodevelopment outcomes. Delay of hippocampal maturation in an extremely preterm group has been reported [50]; thus, hippocampal adequate growth may explain its association with better mental and psychomotor outcomes.

The long-term neurodevelopment evolution of individuals born extremely preterm is, in many cases, more limited as these infants become children. Thus, assessing the long-term neurodevelopment outcomes in children born extremely preterm who undergo neurohabilitation sheds light on the long-term effectiveness of this intervention. A distinctive feature of Katona neurohabilitation is its early administration which is directed to constrain the brain injury evolution and to prevent the subsequent dysmaturational events that result from this injury. The later treatment starts, the smaller the plasticity and the larger the brain damage become [17]. Another relevant characteristic of neurohabilitation is that it requires intensive practice and is administered by the primary care provider; thus, good treatment adherence is imperative. Herein, a direct relation between the number of Katona neurohabilitation follow-up sessions attended and neurodevelopment outcomes was found. Therefore, strategies to improve long-term treatment adherence, such as providing emotional support and parenting training, need to be implemented to consequently enhance neurodevelopmental outcomes.

According to the World Health Organization, there are five adherence dimensions to address to improve treatment results: health system, social–economic, condition-related, patient-related and therapy-related factors [15]. A recent study performed by the Social Work area of the Neurodevelopment Research Unit found that the primary care providers with adequate treatment adherence shared the following characteristics: had the support of family and social networks, planned their activities and routines in advance, got involved in performing the necessary lifestyle changes to complete the treatment and evaluations, and were interested on learning about their offspring’s condition and expected results from attending the diagnostic and therapeutic sessions and practicing at home [51].

### Limitations and Future Directions

The sample size was small because the number of extremely preterm infants is lower. The survival rate of extremely preterm infants is lower than that of term-born and other preterm infants. Upon survival, their discharge from the hospital takes longer due to multiple morbidities which in many cases prevent them from being candidates to this treatment, e.g., when the neurohabilitation maneuvers may compromise their life due to complications with a cardiac disease. Also, the ages at scanning were not uniform though appointments for MRI scanning at specific ages were set. MRI scanning was performed during physiological sleep, which made successful scanning more difficult, thus requiring an average of more than two appointments to successfully obtain brain MRI. Also, respiratory and digestive disease in the infants and children resulted in appointment cancelation, delaying brain MRI acquisition. Therefore, the variability rendered by the latter was accounted for by considering the age at the first scan and the time between scans. To avoid normality assumptions, non-parametric tests were used. The use of volumes relative to the intracranial volume allowed us to control for each subject head size, which is not only a function of individual difference but also depends on the individual’s age and growth rate. Sex was introduced as a control variable for the multiple correlation analysis because there are well-known differences in brain volumes between male and female participants. Also, the hierarchical regression analyses were performed considering first age at first scan, time between scans, sex, and treatment adherence, before subcortical relative volumes were introduced as predictors.

Future studies using larger sample sizes are required to confirm and enrich the findings presented in this work. In addition, future research should evaluate the treatment adherence dimensions for participants and quantitatively compare the neurodevelopment outcomes at different ages at treatment initiation.

## 5. Conclusions

The Katona neurohabilitation treatment showed an adherence-dependent effect on mental and psychomotor neurodevelopment in extremely preterm children. This result was accompanied by discrete increases in most of the subcortical volumes, which resulted in decreased relative volumes, as expected considering the ongoing cortical and white matter maturation. The hippocampal volumes after treatment showed associations with the neurodevelopment outcomes, thus suggesting further research on the association between long-term hippocampal development and cognitive outcomes in extremely preterm children treated with Katona neurohabilitation therapy during infancy is required.

## Figures and Tables

**Table 1:** Prenatal and perinatal risk factors of the extremely preterm participants.

	Measure	Values
Maternal Characteristics		
Infections	*n* (%)	10 (66.7)
Early membrane rupture	*n* (%)	8 (53.3)
Pre-eclampsia	*n* (%)	4 (26.7)
Placental alterations	*n* (%)	3 (20.0)
Intrauterine growth restriction	*n* (%)	2 (13.3)
Diabetes	*n* (%)	1 (6.7)
Infant Characteristics		
Number of days in hospital	Mean (SD)	67.1 (21.3)
Number of days on ventilation	Mean (SD)	40.9 (37.4)
Neonatal sepsis	*n* (%)	13 (86.7)
Hypoxic–ischemic encephalopathy	*n* (%)	9 (60.0)
Intraventricular hemorrhage	*n* (%)	9 (60.0)
Congenital heart disease	*n* (%)	8 (53.3)
Retinopathy of prematurity	*n* (%)	6 (40.0)
Anemia	*n* (%)	5 (33.3)
Bronchopulmonary dysplasia	*n* (%)	5 (33.3)
Necrotizing enterocolitis	*n* (%)	4 (26.7)
Seizures	*n* (%)	3 (20.0)

SD, standard deviation.

**Table 2:** Differences between subcortical absolute volumes in extremely preterm children at the beginning of and after Katona neurohabilitation.

Structure	Hemisphere	Treatment Beginning	After Treatment	z	*p*	Change
		Median (Range)	Median (Range)			
Age at Scan (weeks)		27 (79)	157 (367)			
Amygdala	Left	717 (726)	1181 (848)	−3.408	**0.001**	**↑**
	Right	570 (843)	1351 (1432)	−3.408	**0.001**	**↑**
Caudate	Left	1947 (2146)	2949 (2037)	−3.408	**0.001**	**↑**
	Right	2017 (2222)	3053 (2997)	−3.408	**0.001**	**↑**
Hippocampus	Left	1599 (1990)	3126 (2244)	−3.408	**0.001**	**↑**
	Right	1698 (1683)	3206 (2129)	−3.408	**0.001**	**↑**
Pallidum	Left	1749 (2101)	1394 (1024)	−1.647	0.100	**↓**
	Right	1535 (1482)	1384 (927)	−1.590	0.112	**↓**
Putamen	Left	3140 (2276)	3999 (2777)	−3.408	**0.001**	**↑**
	Right	3175 (2467)	4110 (1824)	−3.408	**0.001**	**↑**
Thalamus	Left	5831 (3266)	5477 (5081)	−0.511	0.609	**↓**
	Right	5775 (3428)	5584 (4845)	−0.341	0.733	**↓**
Subcortical Gray Matter		33,402 (19,621)	44,741 (31,878)	−3.408	**0.001**	**↑**
Cortex		289,075 (357,519)	493,793 (217,370)	−3.408	**0.001**	**↑**
Cerebral White Matter		118,670 (221,699)	260,553 (227,569)	−3.408	**0.001**	**↑**
Intracranial *		470,838 (792,745)	1,052,688 (644,737)	−3.408	**0.001**	**↑**

Wilcoxon signed-rank tests were used (*p* < 0.05 are shown in bold characters). *, the total intracranial volume is included as the reference volume. **↑**—increase, **↓**—decrease.

**Table 3:** Differences in subcortical relative volumes in extremely preterm children at the beginning of and after Katona neurohabilitation.

Structure	Hemisphere	Beginning of Treatment	After Treatment	z	*p*	Change
		Median (Range)	Median (Range)			
Amygdala	Left	0.00153 (0.00206)	0.00116 (0.00051)	−2.045	**0.041**	**↓**
	Right	0.00117 (0.00197)	0.00128 (0.00104)	−1.022	0.307	**↑**
Caudate	Left	0.00329 (0.00204)	0.00254 (0.00165)	−3.181	**0.001**	**↓**
	Right	0.00376 (0.00271)	0.00272 (0.00188)	−2.726	**0.006**	**↓**
Hippocampus	Left	0.00335 (0.00297)	0.00281 (0.00135)	−1.590	0.112	**↓**
	Right	0.00277 (0.00258)	0.00287 (0.00141)	−1.079	0.281	**↑**
Pallidum	Left	0.00443 (0.00626)	0.00130 (0.00068)	−3.010	**0.003**	**↓**
	Right	0.00363 (0.0460)	0.00129 (0.00047)	−3.294	**0.001**	**↓**
Putamen	Left	0.00635 (0.00622)	0.00388 (0.00192)	−2.953	**0.003**	**↓**
	Right	0.00628 (0.00536)	0.00405 (0.00146)	−2.840	**0.005**	**↓**
Thalamus	Left	0.01261 (0.01432)	0.00549 (0.00266)	−3.408	**0.001**	**↓**
	Right	0.01097 (0.01515)	0.00507 (0.00244)	−3.408	**0.001**	**↓**
Subcortical Gray Matter		0.07292 (0.06170)	0.04187 (0.01173)	−2.442	**0.015**	**↓**
Cortex		0.51719 (0.25186)	0.40221 (0.11938)	−3.408	**0.001**	**↓**
Cerebral White Matter		0.21410 (0.19096)	0.24257 (0.09671)	−1.931	0.053	**↑**

Wilcoxon signed-rank tests were used (*p* < 0.05 are shown in bold characters). Relative volumes were obtained by dividing the absolute volume of each subcortical structure by the corresponding total intracranial volume. **↑**—increase, **↓**—decrease.

**Table 4:** Hierarchical regression analyses for MDI and PDI.

Evaluation	Models	R^2^	*p*	Predictor	Beta	*p*
MDI	1	0.415	0.010	Treatment adherence	0.644	0.010
	2	0.598	0.004	Treatment adherence	0.649	0.004
				Right hippocampus relative volume	0.428	0.037
PDI	1	0.319	0.028	Treatment adherence	0.565	0.028
	2	0.543	0.009	Treatment adherence	0.454	0.043
				Left hippocampus relative volume	0.486	0.032

Age at first scan, time between scans, sex, and treatment adherence were introduced stepwise in Block 1. Then, the subcortical relative volumes (Block 2) were added stepwise. The subcortical relative volumes were obtained by dividing the absolute volume by the total intracranial volume. Both relative volumes correspond to MR images obtained after treatment. MDI, mental development index; PDI, psychomotor development index. *p* < 0.05.

## Data Availability

The raw data supporting the conclusions of this article will be made available by the authors on request.

## Supplementary Materials

The following supporting information can be downloaded at https://www.mdpi.com/article/10.3390/brainsci14100957/s1: Figure S1: Age at MRI scan in the extremely preterm individuals; Figure S2: Representative segmentations of the subcortical structures in a participant of this work; Figure S3: Bayley’s Mental (MDI) and Psychomotor (PDI) development scores are associated with the number of treatment follow-up sessions attended; Figure S4: Estimated total intracranial volume (in mm^3^) at the corresponding scan age (in weeks); Table S1: Non-parametric correlations controlled by sex.
