# Supplementary material for: Subcortical Change and Neurohabilitation Treatment Adherence Effects in Extremely Preterm Children
Source: Brain Sci. 2024 Sep 25;14(10):957. doi: 10.3390/brainsci14100957 (PMC11506661; doi:10.3390/brainsci14100957)
Supplement: Supplementary file 1 [file brainsci-14-00957-s001.zip › brainsci-3156967-supplementary.pdf]

SUPPLEMENTARY MATERIALS

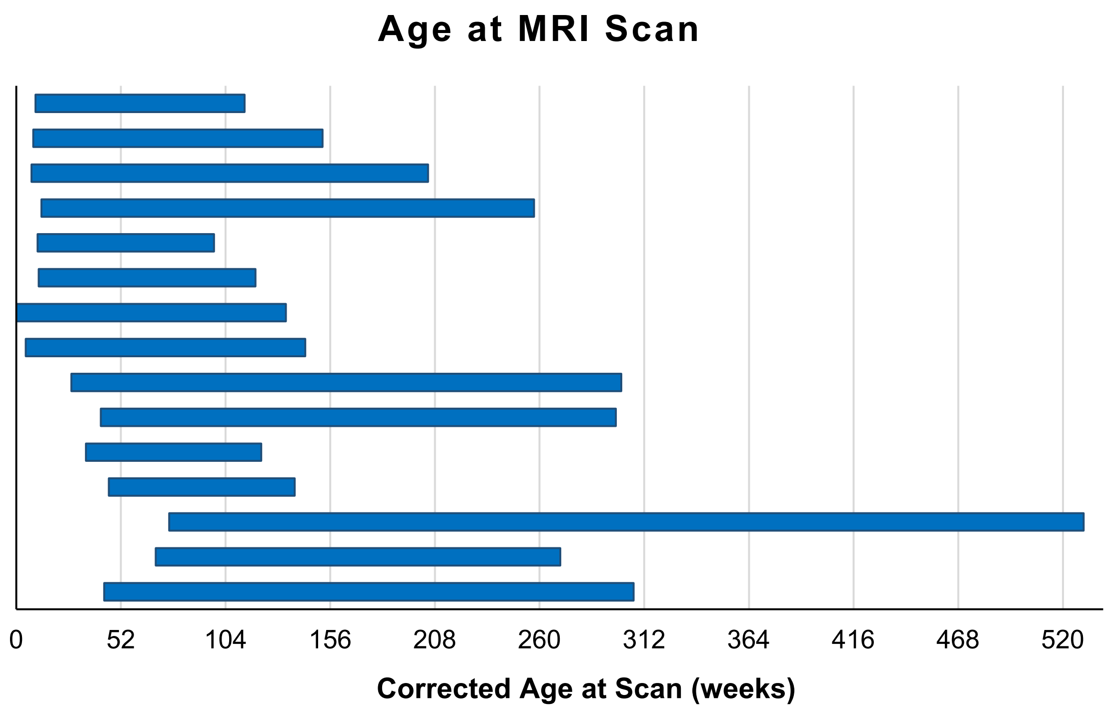

Figure S1. Age at MRI scan in the extremely preterm individuals. Every bar represents a child. The beginning of the bar represents the age at the brain scan acquired at the beginning of the treatment, and the end represents the age at the scan after treatment.

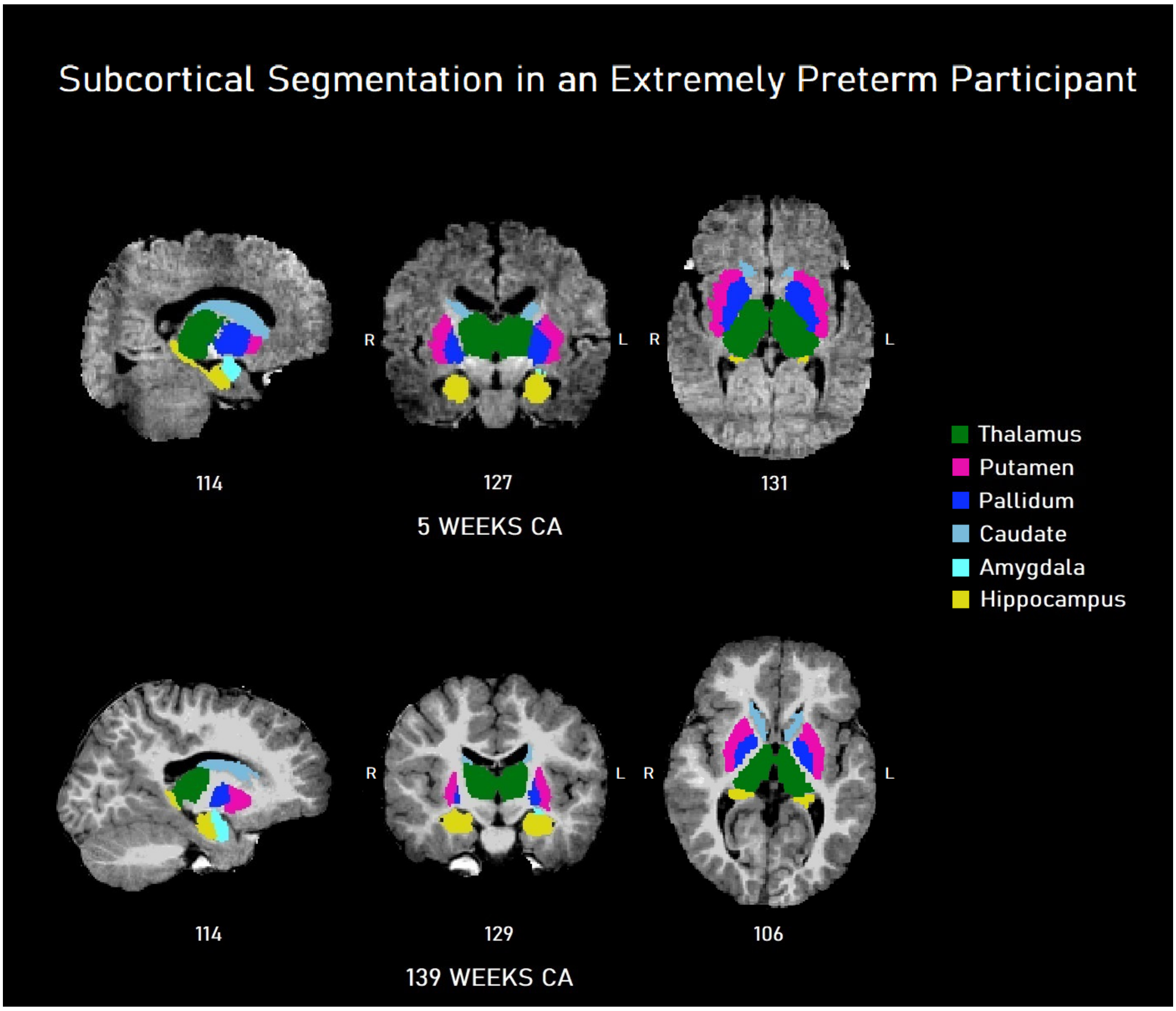

Figure S2. Representative segmentations of the subcortical structures in a participant of this work. CA, chronological age.

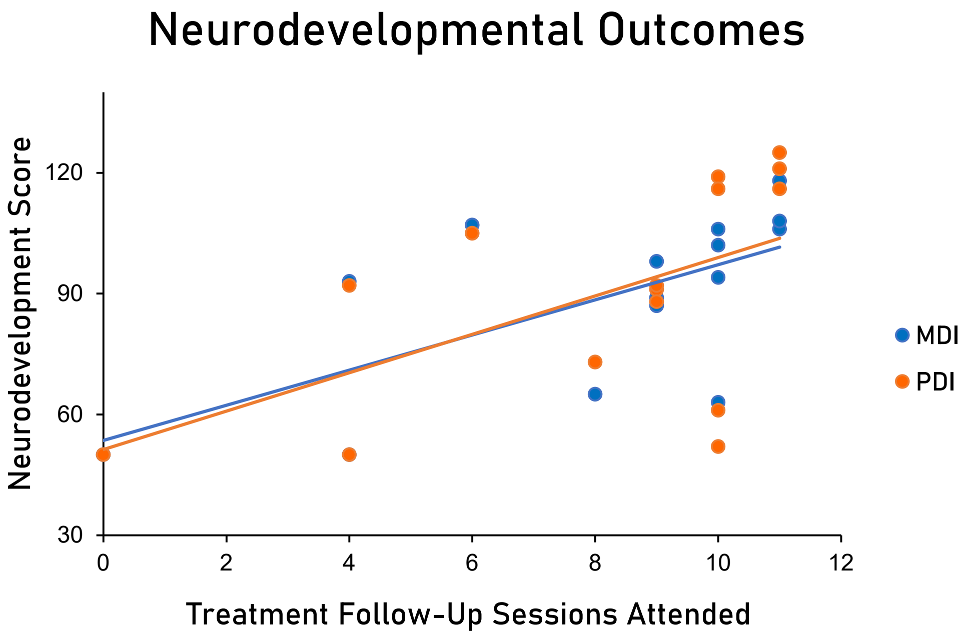

Figure S3. Bayley's Mental (MDI) and Psychomotor (PDI) development scores are associated with the number of treatment follow-up sessions attended.

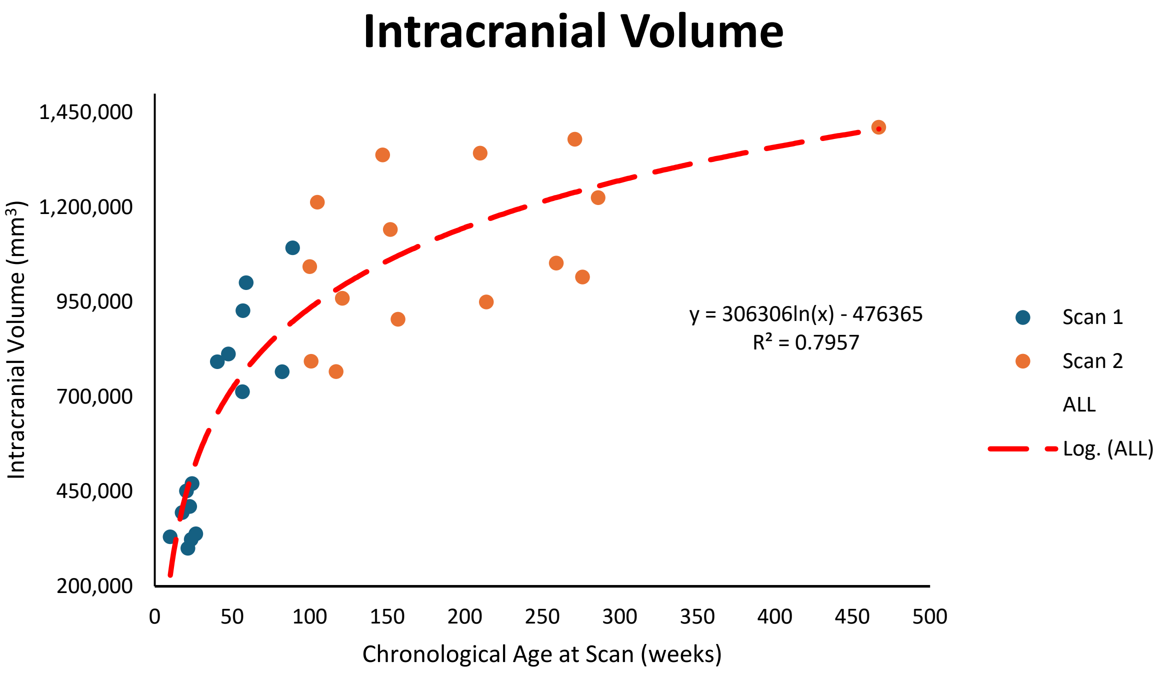

Figure S4. Estimated total intracranial volume (in mm3) at the corresponding scan age (in weeks). Brain scans at the beginning of treatment are represented in blue and after-treatment scans in orange. The logarithmic function showed the best adjustment to model the data distribution (red dashed line).

Table S1. Non-parametric correlations controlled by sex

Measurement Time Point	Hemi	Variable	Tx Adherence		Age at Scan 1		Time b/Scans		MDI		PDI	
			R	p	R	p	R	p	R	p	R	p
**Measurement Time Point**	**Hemi**	*Age at Scan 1*	0.537	0.047	-	-						
		Time b/Scans					-	-	0.589	0.027	0.547	0.043
		MDI	0.653	0.011			0.589	0.027	-	-	0.813	**0.000**
		PDI	0.644	0.013			0.547	0.043	0.813	**0.000**	-	-
**Beginning of Treatment**	**L**	*Amygdala*			-0.703	**0.005**						
		Caudate										
		Hippocampus			-0.641	0.014						
		Pallidum			-0.857	**0.000**						
		Putamen			-0.814	**0.000**					-0.589	0.027
		Thalamus			-0.910	**0.000**						
	**R**	*Amygdala*			-0.669	**0.009**						
		Caudate										
		Hippocampus			-0.673	**0.008**						
		Pallidum	-0.604	0.022	-0.793	**0.001**						
		Putamen			-0.727	**0.003**						
		Thalamus			-0.835	**0.000**						
		Subcortical Gray Matter			-0.818	**0.000**						
		Cortex	-0.553	0.040								
		White Matter	0.758	**0.002**					0.556	0.039		
**After Treatment**	**L**	*Amygdala*										
		Caudate										
		Hippocampus										
		Pallidum										
		Putamen										
		Thalamus										
	**R**	*Amygdala*										
		Caudate										
		Hippocampus										
		Pallidum										
		Putamen					-0.615	0.019				
		Thalamus										
		Cortex					-0.621	0.018				

Note: Hemi, hemisphere; MDI, Bayley's mental development index; PDI, Bayley's psychomotor development index; L, left; R, right; Time b/Scans, time between scans; Tx Adherence, treatment adherence. p < 0.01 is highlighted using bold characters.
